# Upcycling Strategies to Improve the Nutritional Value of Staple Food

**DOI:** 10.3390/foods15040620

**Published:** 2026-02-09

**Authors:** Chiara Russo, Matteo Alessandro Del Nobile, Amalia Conte

**Affiliations:** 1 Department of Social Sciences, University of Foggia, 71122 Foggia, Italy; chiara.russo@unifg.it; 2 Department of Economics, Management and Territory, University of Foggia, 71122 Foggia, Italy; 3 Department of Humanistic Studies, Letters, Cultural Heritage, Educational Sciences, University of Foggia, Via Arpi 176, 71121 Foggia, Italy; amalia.conte@unifg.it

**Keywords:** agri-food by-products, dehydration, sustainability, pasta, gnocchi, fortification

## Abstract

This study investigates four agri-food by-products from broccoli, artichokes, asparagus, and pumpkin, processed into powders through either an industrial or a lab-scale drying and milling process. The resulting powders were evaluated for their nutritional characteristics, revealing that industrial processing generally produced higher-quality powders, likely due to improved moisture removal and reduced thermal damage. Consequently, the four industrial powders were selected for use in the fortification of pasta and gnocchi, which were then analyzed for their nutritional profile in terms of total polyphenols, flavonoids, antioxidant activity, and dietary fiber content. To facilitate a comprehensive comparison, a global quality index (*GQI*) was developed to integrate the different parameters. The index accounted not only for the nutritional enhancement provided by each by-product but also for the potential sensory drawbacks associated with fortification, such as color changes, texture modifications, or flavor impacts. This dual weighting allowed for a balanced evaluation of feasibility and acceptability. The *GQI* enabled the identification of artichoke as the most suitable by-product for each fortified food matrix, as well as gnocchi, between the two products, as the best overall response to fortification. This approach provides a structured method for selecting optimal by-product ingredients and offers valuable insights for future upcycling strategies aimed at improving the nutritional quality of staple foods.

## 1. Introduction

In recent years, there has been a significant and growing emphasis on conscious and healthy dietary habits among global consumers. In this context, the interest in bioactive compounds recovered from agri-food by-products has increased [1]. The trend reflects a commitment to balancing two key objectives: the formulation of health-promoting food products and the sustainability through circular economy practices, able to reduce food loss and minimize the environmental impact [2]. The properties of agri-food by-products depend on the type of vegetable; however, they generally share several common characteristics: they are rich in dietary fiber and antioxidant polyphenolic compounds, including flavonoids, carotenoids, phenolic acids, and anthocyanins, which can act in the body eliminating free radicals and protecting against oxidant stress; in addition, a significant concentration of essential minerals and vitamins are present [3,4].

Generally, by-products have a high-water content and heavy organic load, making them prone to rapid spoilage [5]; consequently, to enhance their stability and extend the shelf life, they are typically processed using dehydration and grinding to produce a powder for optimal preservation and easier incorporation into various formulations. Thermal treatments can significantly influence the drying behavior of by-products, affecting both process efficiency and final material properties. When heat is applied, moisture removal accelerates due to increased evaporation rates, improved internal water diffusion, and partial structural modification of the matrix. These changes can alter texture, porosity, color, and nutrient stability [6]. For example, higher temperatures may enhance drying speed but also increase the risk of thermal degradation or the formation of undesirable volatile compounds [7,8]. However, using high temperatures for short processing times offers several advantages compared with using lower temperatures for longer durations, making the industrial-scale process more attractive than a pilot-scale alternative. Brief exposure to elevated temperatures can minimize unwanted side reactions or degradation that might occur during prolonged heating, thereby helping maintain product quality [9]. Although peak temperatures may be higher, the total energy consumption can be lower because the system is not kept hot for extended periods. On the other side, lab-scale dryers typically allow for precise control over temperature, airflow, and humidity, enabling detailed investigation of drying kinetics and material transformations [10,11]. Because sample quantities are smaller and heat transfer is more uniform, the results often show faster and more homogeneous moisture removal. Nevertheless, these conditions may not fully represent real process variability. While lab-scale studies help understand fundamental mechanisms, industrial implementation is generally designed to handle rapid heating and cooling efficiently, allowing for better energy utilization [12]. The literature offers limited information comparing the effects of industrial thermal treatments with lab-scale processes on plant-based by-products. This gap makes it difficult to assess how processing scale influences product quality, functionality, or stability. Consequently, further research is needed to better understand these differences and to guide the development of more efficient and reliable valorization strategies.

The applications of by-products are more abundant in food, and, in particular, in pasta [13,14,15,16]. Based on evidence from the literature, we can state that products enriched with plant-based by-products often present a clear trade-off: they tend to offer superior nutritional value while exhibiting a decline in overall sensory quality [17]. The additional fiber, micronutrients, and bioactive compounds derived from these by-products can substantially enhance the product’s health profile. However, their inclusion frequently affects taste, texture, and appearance, making the final product less appealing to consumers. The central challenge, therefore, is to find the right balance between nutritional improvement and sensory acceptability. Despite increasing interest in upcycled ingredients and sustainable reformulation, there is currently no validated approach capable of determining the optimal amount of by-product to include. Existing studies often rely on empirical testing or case-specific formulations, without providing a generalizable method for evaluating how much by-product can be added before the product becomes sensorially unacceptable.

To address this gap, this study focused on a comparison of four by-products, pumpkin, asparagus, artichokes, and broccoli, processed by both an industrial and lab-scale dehydration system to be applied in powder to pasta and gnocchi. Currently, there are no officially established recommended intake values or regulatory thresholds specifically for total phenolic compounds, total flavonoids, or antioxidant activity in pasta products. These parameters are generally considered qualitative or comparative indicators of the presence of bioactive compounds rather than mandatory nutritional requirements. For this reason, they are commonly used in research studies to evaluate the functional potential of reformulated foods and to compare the impact of different fortifying ingredients. In the present work, these parameters were therefore calculated for pasta and gnocchi enriched with by-products to quantify the nutritional enhancement achieved through their incorporation and to identify which by-products and product matrices provided the greatest functional improvement. These indices allowed for a meaningful comparison among formulations and supported the assessment of the valorization potential of the selected by-products as sources of health-promoting compounds. The proposed approach introduced a quality index designed to weigh the nutritional benefits of by-product incorporation against the associated sensory drawbacks. This index aimed to provide a more systematic and quantitative framework for decision-making, helping formulators identify the most suitable level of enrichment in each product and the best food to fortify. By integrating both nutritional and sensory dimensions into a single metric, the method offers a practical tool for guiding product development in a more efficient and transparent way.

## 2. Materials and Methods

### 2.1. Raw Materials

Four different vegetable by-products from artichokes, asparagus, broccoli, and pumpkins were provided by the company FARRIS s.r.l. (Foggia, Italy) in two different forms, as fresh by-products to be processed in the lab and as fine powder. Both fresh and powder by-products refer to artichoke stems, leaves, and bracts; asparagus stems; broccoli leaves and stems; and pumpkin peels. The powders were transported to the lab and stored under refrigerated conditions before their use. To obtain the powder, the company used an air-drying process with a continuous tunnel dryer (CMA Food Machinery s.r.l., Salerno, Italy). Before drying, the by-product was subjected to three short consecutive washing steps in interconnected stainless-steel tanks. The washed by-products were then cut into cubes and transferred to the tunnel for dehydration. The overall drying process lasted approximately 3–4 h, with operating temperatures ranging between 70 and 80 °C. After dehydration, the dried vegetable material was ground using an industrial mill (TecnoFrutta s.r.l., Foggia, Italy). During milling, the product temperature was carefully maintained below 35 °C to prevent any potential thermal degradation of thermolabile compounds.

In the lab, each fresh by-product underwent a standardized processing step: washing with water to remove any solid residue, immersion in chlorinated water (20 mL/L) for 5 min, rinsing again with water, and air-drying. The dehydration step was carried out in a hot air conventional dryer (PF–SICCO80PRO, SICCOTECH, Campobasso, Italy), at 60 °C and 5% RH for about 30 h. The dried by-products were milled to reach a fine powder (<500 μm) using two types of lab grinders (16/BV-Beccaria s.r.l., Cuneo, Italy; and Swing type CGOLDENWALL, New York, NY, USA). The resulting powders were stored in plastic bags at 4 °C until further use.

### 2.2. Raw Material Water Content

Both fresh and dehydrated by-products, processed with the industrial plant and the lab-scale equipment, were analyzed for water content (%) using a thermal balance (Sartorius, Göttingen, Germany). Five grams of the sample was distributed uniformly on an aluminum plate and placed in the thermal balance set at 130 °C. For each sample, five replicates were measured.

### 2.3. Pasta and Gnocchi Production

Three ingredients were used to produce fresh pasta (troccoli): durum wheat semolina (1035 g), tap water (270 g), and fresh eggs (195 g). Semolina and fresh eggs were bought from a local market in Foggia (Italy). The ingredients were mixed in an extruder (Monferrina P3, Lineapasta, Italy). For the fortified sample, a different amount of by-product was first hydrated with water and then added to the remaining dough in the extruder tank. After proper mixing for about 10 min, the dough was extruded, obtaining pasta in the form of troccoli. Table 1 reports the weight percentage of each ingredient to make pasta.

The ingredients used to prepare the gnocchi were tap water, potato flakes, wheat flour, potato starch, salt, lactic acid, sorbic acid, and safflower oil. The weighed water was added to the hopper of the gnocchi machine (Condor, Italy), heated to 130 °C, and once this temperature was reached, the heating was stopped, and the rest of the ingredients were added. After mixing for about 15 min, the dough was manually shaped and formed into individual gnocchi pieces. For the fortified samples, all the ingredients were partially substituted with each by-product, previously hydrated with water, and also using carboxymethyl cellulose (CMC), previously dissolved in the water. The remaining production steps were identical to the control gnocchi, with the dough mixing occurring for 15 min before manual shaping. The weight percentage of ingredients in the gnocchi samples is reported in Table 2.

The optimization criterion employed in the present study for the formulation of by-product-enriched gnocchi and pasta was established as follows: For each formulation, the weight fraction of ingredients was systematically adjusted to maximize the incorporation of the by-product while maintaining the overall quality of the cooked product within the target range of 6 to 7, which meant full acceptability. The overall quality of the cooked sample was selected as an optimization parameter, as it consistently exhibited a lower score than the corresponding raw sample. This meant that a preliminary optimization process was carried out in our lab before the current study, with both pasta and gnocchi to identify the concentration of each by-product for the two foods (data not published). Egg pasta was selected instead of egg-free pasta because its intrinsic composition offered greater structural stability when incorporating alternative ingredients such as plant-based powder. In fact, ovalbumin enhances dough cohesiveness, elasticity, and binding capacity, helping to counterbalance the potential weakening of the gluten network. Eggs also contribute to better cooking performance and reduce the risk of excessive cooking loss often observed in enriched formulations.

### 2.4. Nutritional Analyses

The following chemicals were used for the analyses: methanol, Folin–Ciocalteu reagent, gallic acid monohydrate, anhydrous sodium carbonate, methanol, hydrochloric acid, 2,2-azino-bis (3-ethylbenzothiazoline-6- sulfonic acid) diammonium salt (ABTS), potassium persulfate, Trolox (6-hydroxy-2,5,7,8- tetramethylchroman-2-carboxylic acid), aluminum chloride, sodium nitrite, sodium hydroxide solution, and quercetin, supplied by Sigma Aldrich (Milan, Italy). Anhydrous sodium carbonate was obtained from Carlo Erba (Milan, Italy). All the reagents used were of analytical grade.

Total phenols, total flavonoids, antioxidant activity, and fiber content were assessed for each by-product in the form of powder and for the final fortified samples (pasta and gnocchi with by-products). For the fortified samples, the extraction for the chemical analyses was performed as described by Panza et al. [18], with slight modifications. First, the fortified samples were dried with a ventilated stove (BINDER GmbH, Tuttlingen, Germany) at 40 °C for 20 h and milled to obtain powder. Briefly, 2 g of powder was mixed with 20 mL of methanol aqueous solution (80:20), included in 50 mL centrifuge tubes, homogenized, subjected to ultrasound treatment for 15 min, according to Natrella et al. [19], and centrifuged at 4 °C for 10 min at 10,000 rpm (5804R, Eppendorf, Milan, Italy). Then, each extract was collected and filtered (PTFE, 0.45 µm) and used for the analytical determinations. All the extractions were made in triplicate, with appropriate dilutions. The total phenolic content (TPC) was determined by the Folin–Ciocalteu method, as described by Panza et al. [18], and expressed as milligrams of gallic acid equivalents (GAE) per gram of dry weight (dw), according to a calibration curve (6.25–300 mg/L; R^2^ = 0.998). The aluminum chloride method, as described by Panza et al. [18], was used for the total flavonoid content (TFC). TFC was expressed in milligrams of quercetin equivalent (QE) per gram of dry weight, according to a calibration curve (25–800 mg/L; R^2^ = 0.996). The antioxidant activity was evaluated by both ABTS and FRAP methods. The results of the ABTS assay, as described by Re et al. [20], were expressed as milligrams of Trolox equivalents (TE) per gram of dry weight, according to a calibration curve (3.25–600 mg/L; R^2^ = 0.992). The results from the FRAP method, as described by Marinelli et al. [21], were expressed as μmol of ferrous equivalent Fe(III) per gram of dry weight, according to a calibration curve (0.0125–1.25 mmol/L; R^2^ = 0.997). All analyses were carried out in triplicate. Total dietary fiber was determined by the AOAC Official Method (985.29) [22]. It was expressed in g/100 g. The analysis was conducted at the NIRO s.r.l. lab (Campobasso, Italy).

### 2.5. Sensory Analyses

For sensory testing, pasta and gnocchi samples were served to a panel of nine well-trained judges (5 female and 4 males, ages ranging from 30 to 50 years). A quantitative descriptive analysis (QDA) was used for sample comparison, according to the guidelines of the Codex Alimentarius Commission and standards of ISO 13299:2016 [23]. Panelists had a long testing experience in sensory evaluation of pasta, but they were re-trained in two subsequent sessions (1 h/day for 2 days) to align their judgments and define the sensory parameters. During the test, they were asked to indicate odor, color, homogeneity, breaking strength, and appearance of fresh raw samples and odor, color, elasticity, firmness, and taste of fresh cooked troccoli. In addition, each member was asked to score the overall quality of both raw and cooked pasta. For uncooked gnocchi, the panelists evaluated four key attributes: color, odor, homogeneity, and overall quality. For cooked gnocchi, a different set of attributes was assessed: color, odor, adhesiveness, grittiness, taste, and overall quality. For the evaluation, a 9-point scale was used (1 = lowest score; 9 = highest score; 5 = threshold of acceptability). The sensory evaluation was conducted over two separate sessions: one for pasta and another for gnocchi, each lasting about 1 h. Samples were prepared following good manufacturing practices, under identical conditions, and presented to the panelists within a controlled environment. To ensure objectivity, all samples were served anonymously, in randomized order, using three-digit coded containers. All samples were presented at the same temperature and in equal portions. Panelists were instructed to rinse their mouths with water between each sample to cleanse the palate and minimize carry-over effects. An appropriate protocol was followed to protect the rights and privacy of the panelists. This included obtaining verbal informed consent, ensuring participation was voluntary, and allowing participants the ability to withdraw at any time, as well as the full disclosure of study requirements and risks, and not releasing participant data without their knowledge.

### 2.6. Calculation of the Global Quality Index

The procedure used by Lordi et al. [24] was also used to calculate the Global Quality Index (GQI). The positive aspects associated with fortification were related to the increased nutritional quality (such as total dietary fiber, FRAP, and ABTS), while the negative aspect associated with fortification was the sensory quality.

The normalization of the quality indices was conducted according to the following expressions:

(1) $$Normalized\;Total\;Dietary\;Fiber=\;\frac{{PQI}_{TDF}^{Act}\;-\;{PQI}_{TDF}^{CTR}}{{PQI}_{TDF}^{CTR}}\cdot 100$$

(2) $$Normalized\;FRAP=\frac{{PQI}_{FRAP}^{Act}-{PQI}_{FRAP}^{CTR}}{{PQI}_{FRAP}^{CTR}}\cdot 100$$

(3) $$Normalized\;ABTS=\frac{{PQI}_{ABTS}^{Act}-{PQI}_{ABTS}^{CTR}}{{PQI}_{ABTS}^{CTR}}\cdot 100$$

(4) $$Normalized\;Sensory\;Quality=\frac{{NQI}_{SQ}^{CTR}-{NQI}_{SQ}^{Act}}{{NQI}_{SQ}^{CTR}}\cdot 100$$

where ${PQI}_{TDF}^{CTR}$ is the Quality Index of the control sample related to the Total Dietary Fiber, ${PQI}_{TDF}^{Act}$ is the Quality Index of the active sample related to the Total Dietary Fiber, ${PQI}_{FRAP}^{CTR}$ is the Quality Index of the control sample related to FRAP, ${PQI}_{FRAP}^{Act}$ is the Quality Index of the active sample related to FRAP, ${PQI}_{ABTS}^{CTR}$ is the Quality Index of the control sample related to ABTS, ${PQI}_{ABTS}^{Act}$ is the Quality Index of the active sample related to ABTS, ${NQI}_{SQ}^{CTR}$ is the Quality Index of the control sample related to the Sensory Quality, and ${NQI}_{SQ}^{Act}$ is the Quality Index of the active sample related to the Sensory Quality. It is worth noting that each one of the normalized quality indices represents the percentage difference between the active sample (i.e., the sample fortified with the investigated by-product) and the control. The GQI was calculated according to the following expression:

(5) $$GQI=\;\frac{\frac{1}{3}\cdot \left(\frac{{PQI}_{TDF}^{Act}\;-\;{PQI}_{TDF}^{CTR}}{{PQI}_{TDF}^{CTR}}\;+\;\frac{{PQI}_{FRAP}^{Act}\;-\;{PQI}_{FRAP}^{CTR}}{{PQI}_{FRAP}^{CTR}}\;+\frac{{PQI}_{ABTS}^{Act}\;-\;{PQI}_{ABTS}^{CTR}}{{PQI}_{ABTS}^{CTR}}\right)}{\frac{{NQI}_{SQ}^{CTR}-{NQI}_{SQ}^{Act}}{{NQI}_{SQ}^{CTR}}}$$

### 2.7. Statistical Analysis

The statistical analysis was performed using JMP Student Edition 18 (SAS Institute Inc., Cary, NC, USA). One-way analysis of variance (ANOVA) was conducted, followed by the Tukey–Kramer HSD test with the option for homogeneous groups (*p* < 0.05), to determine significant differences among the evaluated parameters.

## 3. Results and Discussion

### 3.1. Effect of Processing Conditions on By-Product Properties

Table 3 reports the percentage water content values of fresh and dehydrated by-products, processed both with the industrial plant and lab-scale equipment. As shown by the data reported in the table, the water content of the fresh by-products is very similar, and only in some cases, the differences, although small, are statistically significant (*p* < 0.05). Regarding the dehydrated by-products, it can be observed that those with a higher initial water content retain a higher water content in the dehydrated product as well, although the differences among the various by-products remain small (*p* < 0.05). Concerning the two dehydration processes employed, no specific trend appears to emerge. The differences between the two methods are not always statistically significant (*p* > 0.05), and when they are, the magnitude of the differences is limited. This can be explained by the differences in the structure of vegetables; raw materials characterized by a lower initial moisture content, due to their dense cellular structure and lower hygroscopicity, tend to reach a lower final water content compared to vegetables characterized by high water concentrations [25].

Table 4 reports the main nutritional characteristics of the ingredients used for pasta and gnocchi production. Regarding the by-products, the table includes the values of those dehydrated in the laboratory, as well as those dehydrated using the industrial plant. As evident from the data reported in the table, the main nutritional indices are consistently higher than those of the ingredients used, and the differences are statistically significant (*p* < 0.05), except in the case of pumpkin, only regarding flavonoid content and FRAP. It is worth noting that, regarding the fiber content of CMC, it is much higher than that of all the other ingredients; however, as previously discussed, CMC is used in very small quantities to produce the fortified pasta. Therefore, its contribution to the fiber content can be considered negligible. As shown by the data, differences are observed between the values obtained for the by-products processed in the lab and those produced on the industrial scale. These differences are small and, in several cases, not statistically significant (*p* > 0.05). Nevertheless, in most cases, the industrially produced by-products exhibit higher values. This discrepancy is likely attributable to the different processing conditions. In fact, although higher temperatures were reached in the industrial process (between 70 °C and 80 °C, compared with 60 °C in the lab-scale system), the overall processing time was much shorter (3–4 h compared to 30 h in the lab). The drying temperature significantly impacts the drying rate, which in turn plays a crucial role in preserving the nutritional quality. For instance, this is particularly evident in carotenoid retention, which can be enhanced when a higher drying temperature is coupled with shorter drying times [26]. Indeed, several studies have investigated the influence of processing temperatures on the antioxidant activity of vegetables, frequently observing that higher processing temperatures lead to shorter drying times and maximize the antioxidant activities. This effect is probably attributed to the rapid thermal inactivation of pro-oxidative enzymes during high-temperature treatment, which stops the enzymatic degradation pathways throughout the drying process [27,28]. Regarding the differences among the various by-products investigated, the artichoke exhibited markedly higher values for the main nutritional indicators measured, whereas the pumpkin and broccoli showed the lowest ones. Similar results were documented by Tiveron et al. [29], who showed that the content of phenolic compounds was highest in artichoke compared to the other vegetables (asparagus, broccoli, and pumpkin).

Given that the processing conditions do not significantly affect the nutritional characteristics of the by-products investigated and considering that in most cases the industrially produced by-products exhibit higher values, the industrially produced by-products were used to produce pasta and gnocchi.

### 3.2. Sensory Quality of Pasta and Gnocchi with and Without By-Products

**Figure 1:**
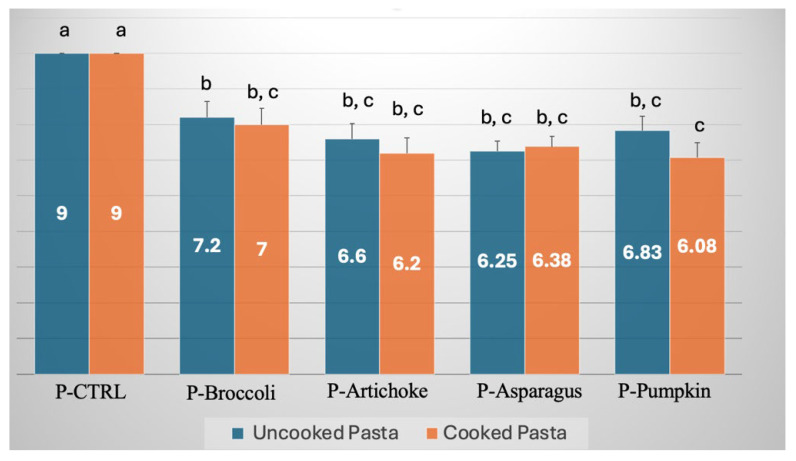
Overall quality of cooked and uncooked pasta with and without by-products. P-CTRL—control pasta; P-Broccoli—pasta with broccoli by-products; P-Artichoke—pasta with artichoke by-products; P-Asparagus—pasta with asparagus by-products; P-pumpkin—pasta with pumpkin by-products. Data with different letters are statistically different (*p* < 0.05).

**Figure 2:**
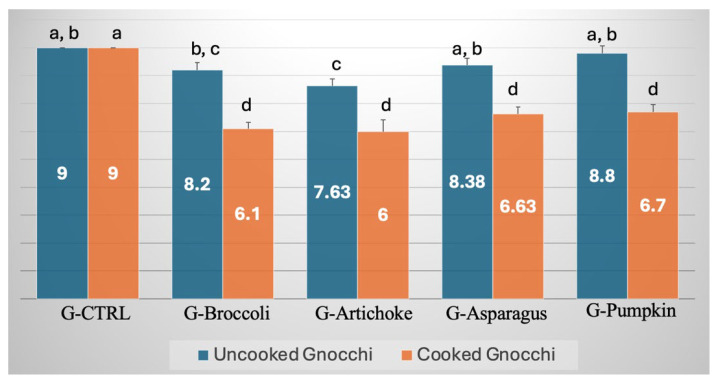
Overall quality of cooked and uncooked gnocchi with and without by-products. G-CTRL—control gnocchi; G-Broccoli—gnocchi with broccoli by-products; G-Artichoke—gnocchi with artichoke by-products; G-Asparagus—gnocchi with asparagus by-products; G-Pumpkin—gnocchi with pumpkin by-products. Data with different letters are statistically different (*p* < 0.05).

Figure 1 and Figure 2 report the overall quality values of pasta and gnocchi, respectively. Each figure shows the quality of both the uncooked and cooked products. As shown by the data reported in the two figures, the overall quality of the cooked samples is consistently lower than that of the corresponding uncooked samples, with the sole exception of pasta enriched with asparagus, although even in this case, the values are very similar, and the differences are not statistically significant (*p* > 0.05). It follows that, between the overall quality of the uncooked and cooked products, the latter represents the limiting factor for product acceptability. This is the reason why the overall quality of the cooked samples was used in the optimization criterion. Regarding the data reported in Figure 1, it can be observed that, except for the control, the differences among all other samples are not statistically significant (*p* > 0.05). Data in Figure 2 show similar trends. It is worth noting that the data presented in these figures are a direct consequence of the optimization criterion adopted in this study. In fact, apart from the control, the overall quality values of both cooked pasta and cooked gnocchi are very close to one another and fall within the range of 6 to 7. For this reason, the overall quality cannot be used as a parameter to assess the sensory impact of each individual by-product on the product quality. Given the criterion adopted for optimizing the formulation, the parameter selected to evaluate the impact of the by-products on sensory quality is the mass percentage of the by-product in the optimized formulation.

**Figure 3:**
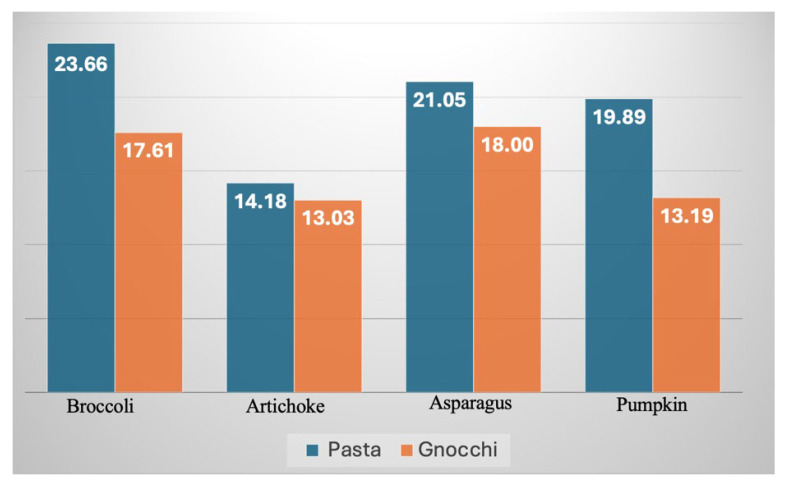
Weight percentage of each by-product in the optimized pasta and gnocchi.

Figure 3 shows the weight percentage of the by-products in the optimized pasta and gnocchi samples. As indicated by the data in the figure, for each by-product, the weight percentage in the fortified pasta samples is always higher than in the corresponding gnocchi samples. This result suggests that pasta is more suitable for fortification, as it can incorporate a higher amount of by-product while maintaining the same overall quality. Regarding the sensory impact of the four by-products studied, it is observed that for pasta, broccoli has the least impact on sensory quality, followed by asparagus and artichoke, with very similar values, and finally, pumpkin. With respect to the fortified gnocchi, broccoli and asparagus have the lowest impact, followed by artichoke and pumpkin. These results are consistent with data from the existing literature, which indicate that enrichment with some of these by-products yields positive sensory quality, with scores exceeding 6 on scales ranging from 1 to 9–10. Regarding pasta with broccoli leaf, Drabrinsska et al. [13] demonstrated a low sensory impact with high powder concentration. Malucelli et al. [30] also reported high positive sensory scores when enriching gnocchi with broccoli residues. Similarly, Szymandera-Buszka et al. [31] found that among cauliflower, broccoli, and pumpkin, the latter was the superior ingredient from a sensory perspective; furthermore, pumpkin was positively evaluated as a pasta additive when incorporated at low concentrations.

### 3.3. Nutritional Quality of Pasta and Gnocchi with and Without By-Products

Table 5 reports the main nutritional indicators (i.e., polyphenol and flavonoid contents, FRAP, ABTS, and TDF) of fortified pasta and gnocchi, together with the values of the corresponding two control samples. The nutritional quality of fortified foods investigated in this work depends both on the inherent characteristics of the by-product used for fortification and on the extent of incorporation (i.e., its weight fraction). Assuming that the contributions of the individual components are linearly additive, as described by the ideal mixture theory, the main nutritional indices of the fortified pasta can be estimated once the weight fractions of its constituents (Figure 3) and their intrinsic properties (Table 4) are known. This estimation holds when interactions among components are negligible, such that the behavior approximates that of ideal mixtures, where interactions between different constituents are equivalent to those occurring among molecules of the same species. It is worth noting that, in the presence of a heterogeneous mixture, the nutritional quality of the food is effectively determined by the linear superposition of the contributions of the individual phases. The predicted values of the main nutritional indicators are reported in Table 5. The goodness of prediction was expressed by the mean relative deviation modulus $\left(\bar{E}\%\right)$, recently reported in Lordi et al. [24]:

(6) $$\bar{E}\%\;=\;\frac{100}{N}\cdot \sum_{i=1}^{i=N}\frac{\left|M_{i}^{exp}-M_{i}^{pred}\right|}{\left|M_{i}^{exp}\right|}$$

where N is the number of experimental data, $M_{i}^{exp}$ is the experimental value, $M_{i}^{pred}$ is the predicted value. Table 5 presents the $\bar{E}\%$ values calculated for each of the nutritional indices examined in this study. As shown by the data reported in the table, the $\bar{E}\%$ values range from a minimum of 1.2 to a maximum of 76.9. Considering the extreme simplicity of the ideal mixtures model used for prediction, it is not the highest $\bar{E}\%$ value that is surprising, but rather the lowest one. Such low $\bar{E}\%$ values were most likely obtained due to imperfect mixing of food components. In such cases, the food behaves as a heterogeneous mixture rather than a homogeneous one. As shown by the data reported in Table 5, both fortified samples of pasta and gnocchi consistently exhibit higher antioxidant activity (i.e., FRAP and ABTS) than their respective controls, although the differences are not always statistically significant (*p* < 0.05). This observation can be attributed to the markedly higher antioxidant activity of the by-products studied, compared with that of the basic ingredients of pasta and gnocchi (Table 4). As expected, the same trend is observed for both polyphenol and flavonoid content, meaning that the fortified samples consistently show higher mean values than their respective controls.

Regarding the differences among the various by-products, for both pasta and gnocchi, it is observed that fortification with artichoke by-products yields the best results in terms of antioxidant activity. This is followed by asparagus and broccoli that are similar, and finally by pumpkin. As expected, the polyphenol and flavonoid contents of the fortified pasta and gnocchi follow a similar trend to that described above. The observations can be attributed both to the characteristics of the individual by-products (Table 4) and to their weight percentage in the fortified samples (Figure 3). The predicted values reported in Table 5 provide an albeit simplistic indication of how these factors combine. As shown by the data in the table, for both pasta and gnocchi, the predicted FRAP and ABTS values follow essentially the same trend as the measured ones.

In line with our findings, Bavaro et al. [32] also demonstrated a higher antioxidant capacity, primarily measured by ABTS and FRAP assays, in pasta enriched with artichoke bracts. Similarly, Vital et al. [33] observed that increasing the concentration of asparagus by-products led to a higher polyphenol content and, more significantly, enhanced antioxidant activity. Comparable trends have been reported regarding the addition of pumpkin seeds to pasta and bread [34].

Previously, the effect of the by-product on the antioxidant activity of fortified foods investigated in the present study was discussed; in that case, pasta or gnocchi were fixed, and the type of by-product was varied. Here, the effect of the fortified matrix on antioxidant activity is discussed. In this analysis, the by-product is fixed, and the matrix is varied. In other words, for each by-product, it will be shown which matrix exhibits the highest antioxidant activity. Given that the comparison is made using the same by-product, one would expect the unique factor influencing the antioxidant activity to be the by-product concentration. According to Figure 3, fortified pasta should exhibit higher antioxidant activity than fortified gnocchi. However, data reported in Table 5 show that this trend is respected only in the case of ABTS; regarding the FRAP values and the polyphenol and flavonoid contents, the exact opposite occurs. Probably, these results can be attributed to the physical structure and the composition of pasta, particularly related to the presence of eggs. Specifically, egg protein, rich in sulfur-containing amino acids and hydrophobic regions, forms hydrogen bonds and hydrophobic interactions with the hydroxyl groups of the polyphenols [35]. Therefore, the proteins can bind with flavonoids, potentially masking their presence and reducing their detectable antioxidant activity, thus provoking an underestimation of the total antioxidant activity in pasta samples.

Regarding fiber content (TDF) of fortified samples, data reported in Table 5 indicate that these products consistently show higher values than their respective controls, with differences that are always statistically significant (*p* < 0.05). This trend was expected, given the higher fiber content of selected by-products [5] compared to the main ingredients of pasta and gnocchi. Regarding the role of by-products, the data in Table 5 show that the same trend observed for antioxidant activity is also found for fiber content; that is, artichoke displays the highest fiber content, followed by asparagus and broccoli, which exhibit similar values, and finally pumpkin. As observed for antioxidant activity, both pasta and gnocchi show the same trend. As previously discussed, the observed trend can be attributed partly to the fiber content of the individual by-products and partly to their relative proportion in the formulation. The high fiber content of artichoke by-products is preserved in the powder form, and therefore, it was also retained after food processing. Examples include gluten-free bread and fresh pasta enriched with artichoke and olive pomace by-products [36,37]. Due to the distinct fiber structure of vegetables, a different approach is typically used for broccoli and asparagus. Iwasssa et al. [38] developed fiber concentrates from asparagus stalks for easier integration into functional foods; likewise, Núñez-Gómez et al. [39] extracted dietary fibers from broccoli stems for similar purposes. As for pumpkin, increasing the powder content in food matrices consequently raises fiber content. However, low concentrations are typically chosen for practical applications [40,41].

Regarding the role of the food matrix, data reported in Table 5 follow the expected trend; that is, fortified pasta consistently shows higher fiber content values than the corresponding fortified gnocchi. This is because the proportion of by-product in pasta is higher than that in gnocchi (Figure 3).

### 3.4. Global Quality Index

**Figure 4:**
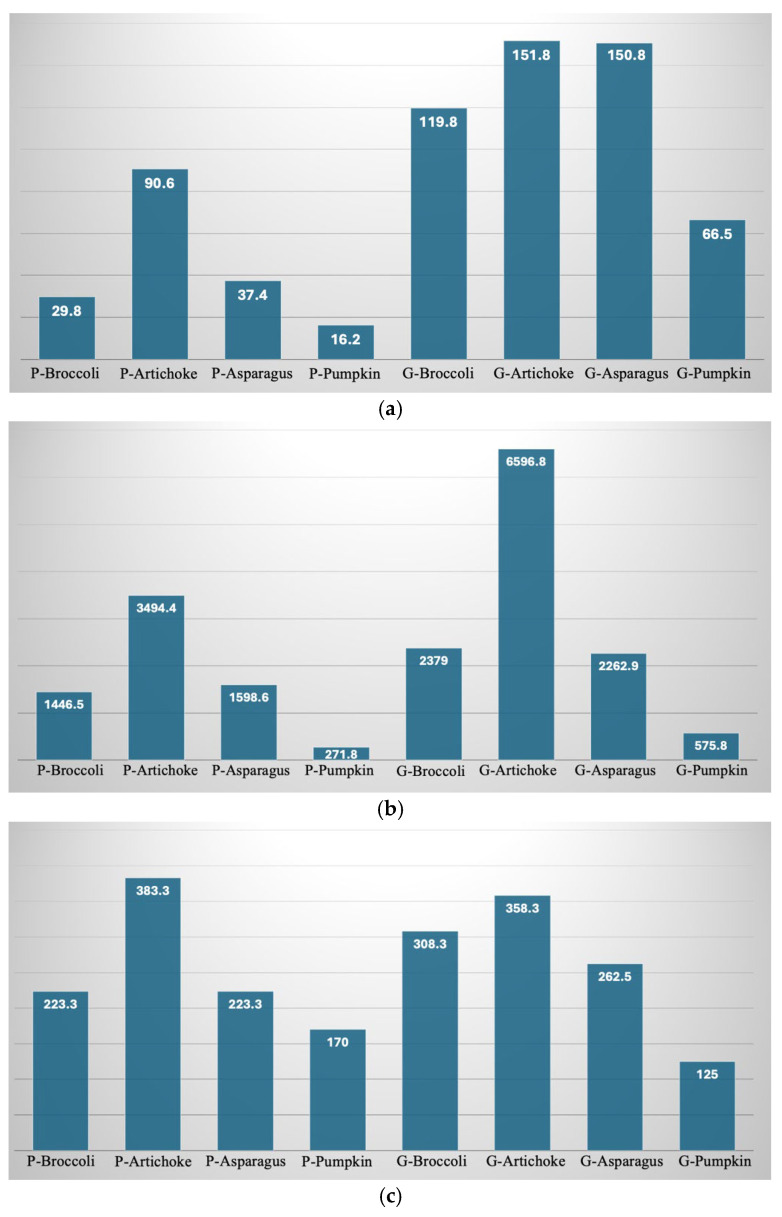
(**a**) Normalized quality index associated with ABTS of pasta and gnocchi fortified with by-products. (**b**) Normalized quality index associated with FRAP of pasta and gnocchi fortified with by-products. (**c**) Normalized quality index associated with total dietary fiber content of pasta and gnocchi fortified with by-products. P-CTRL—control pasta; P-Broccoli—pasta with broccoli by-products; P-Artichoke—pasta with artichoke by-products; P-Asparagus—pasta with asparagus by-products; P-pumpkin—pasta with pumpkin by-products. G-CTRL—control gnocchi; G-Broccoli—gnocchi with broccoli by-products; G-Artichoke—gnocchi with artichoke by-products; G-Asparagus—gnocchi with asparagus by-products; G-Pumpkin—gnocchi with pumpkin by-products.

Figure 4a–c reports the main normalized nutritional indicators. It is worth noting that each individual normalized indicator measures the extent to which the fortification increases the nutritional value compared to the control (non-fortified sample). Therefore, it is inherent in the definition of a normalized indicator that its value depends on both the fortified sample and the control. As shown in Figure 4, each of the normalized indicators is much greater than 1, indicating that the studied by-products markedly enhance the nutritional features of the control. This result is not unexpected considering the data reported in Table 5. The effect of the individual by-products is entirely consistent with what was previously observed; indeed, for both pasta and gnocchi, artichoke is the by-product with the highest values, followed by broccoli and asparagus, which show similar values, and finally pumpkin. The effect of the food matrix shows gnocchi outperforming pasta, except in the case of the normalized indicator related to the fiber content of artichoke and pumpkin.

**Figure 5:**
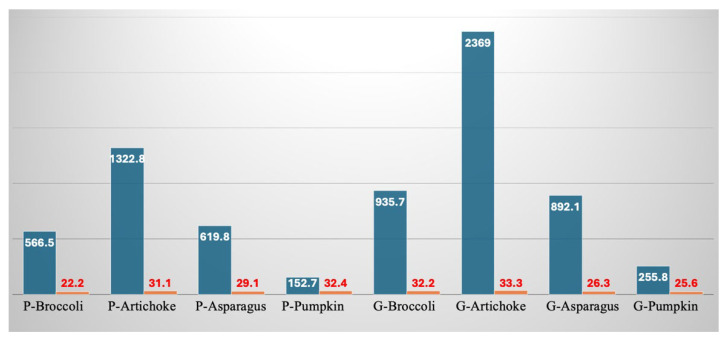
Normalized quality index associated with mean values of nutritional quality (first histogram in blue) and sensory quality (second histogram in orange) of pasta and gnocchi fortified with by-products. P-CTRL—control pasta; P-Broccoli—pasta with broccoli by-products; P-Artichoke—pasta with artichoke by-products; P-Asparagus—pasta with asparagus by-products; P-pumpkin—pasta with pumpkin by-products. G-CTRL—control gnocchi; G-Broccoli—gnocchi with broccoli by-products; G-Artichoke—gnocchi with artichoke by-products; G-Asparagus—gnocchi with asparagus by-products; G-Pumpkin—gnocchi with pumpkin by-products.

Figure 5 reports the mean of the normalized nutritional indicators and the normalized sensory quality, in other words, the numerator and denominator of Equation (5). As can be seen from the data shown in the figure, the numerator is by far greater than the denominator, and this holds for all the by-products investigated and for both food matrices.

**Figure 6:**
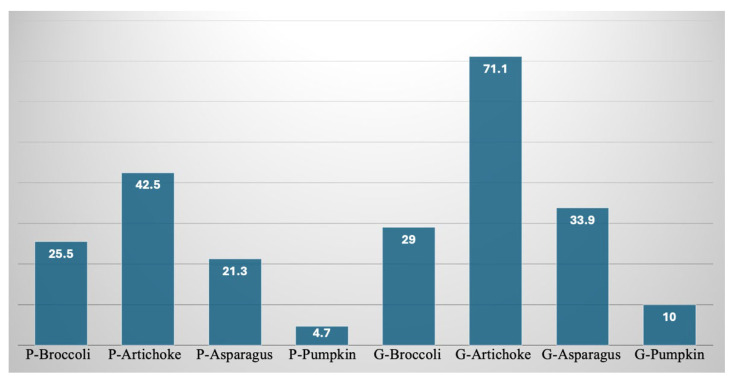
Global quality index calculated from Equation (5) using normalized nutritional and sensory data. P-CTRL—control pasta; P-Broccoli—pasta with broccoli by-products; P-Artichoke—pasta with artichoke by-products; P-Asparagus—pasta with asparagus by-products; P-pumpkin—pasta with pumpkin by-products. G-CTRL—control gnocchi; G-Broccoli—gnocchi with broccoli by-products; G-Artichoke—gnocchi with artichoke by-products; G-Asparagus—gnocchi with asparagus by-products; G-Pumpkin—gnocchi with pumpkin by-products.

Therefore, the data shown in Figure 6**,** reporting the Global Quality Index (GQI) values for all fortified samples are not surprising. Indeed, all GQI values are well above 1, indicating that the positive aspects associated with fortification far outweigh the negative ones. Among the by-products studied, artichoke exhibits the highest GQI, followed by broccoli and asparagus, with similar values, and finally pumpkin. This trend closely mirrors that observed for antioxidant activity and fiber content. Among the two food matrices studied, gnocchi show the highest GQI values. In fact, data presented in Figure 6 indicate that gnocchi fortified with artichoke represent the sample that best balances the positive and negative aspects associated with fortification.

## 4. Conclusions

This study evaluated four agri-food by-products that were dehydrated and milled both on an industrial scale and under controlled laboratory conditions, with the objective of assessing their suitability as functional ingredients for the fortification of pasta and gnocchi. The processing workflow adopted in the two environments ultimately yielded powders with very similar residual moisture contents, demonstrating that industrial dehydration and milling can reliably reproduce laboratory-level conditions. Once obtained, these powders were subjected to a complete nutritional characterization, which revealed modest yet consistent advantages in the samples produced at the industrial level. These results support the potential for scaling up the valorization of such by-products and justify their prospective use as fortifying agents within commercial production chains. The fortified pasta and gnocchi prototypes were developed following preliminary sensory trials designed to maintain overall acceptability scores within the target range of 6–7. Achieving this threshold required different inclusion levels for each by-product, as their sensory impacts varied in intensity and nature. In general, pasta appeared more suitable for fortification than gnocchi, since it tolerated higher levels of powder incorporation without significantly compromising sensory quality. This characteristic suggests that pasta may serve as a more flexible base matrix for introducing nutritionally enhanced ingredients while still meeting sensory expectations. However, when all fortified samples were subjected to comprehensive nutritional analyses, a contrasting tendency emerged. Despite accepting lower amounts of by-products to remain sensorially acceptable, gnocchi displayed higher concentrations of polyphenols and flavonoids, as well as superior antioxidant activity, compared with the corresponding pasta samples. A plausible explanation lies in the masking or binding effects linked to the presence of eggs in the pasta formulations, which may reduce the availability or detectability of bioactive compounds. Conversely, the simpler composition of gnocchi appears to allow for these compounds to remain more active or bioaccessible, thereby enhancing the nutritional profile even at reduced fortification levels. To integrate sensory and nutritional findings into a single evaluation framework, a global quality index was applied, weighting the positive attributes, primarily linked to nutritional improvements, against negative ones associated with sensory penalties. This composite indicator clearly identified artichoke by-product as the most promising fortifying material for both pasta and gnocchi, consistently delivering the most balanced combination of enhanced nutritional value and acceptable sensory performance. Furthermore, when comparing the two fortified food matrices, gnocchi achieved the higher overall score, indicating that it may represent the more suitable product for fortification when both advantages and drawbacks are jointly considered.

Overall, the results demonstrate the feasibility of industrially producing nutritionally enriched powders from food by-products and incorporating them into staple foods without undermining final acceptance. These findings collectively support the broader adoption of by-product valorization strategies as a viable approach to improving the nutritional quality of everyday foods while contributing to circular economy objectives. Future studies could further investigate the technological performance, bioavailability of bioactive compounds, shelf-life stability, and consumer acceptance of fortified pasta and gnocchi, as well as explore different inclusion levels and combinations of by-products to optimize both nutritional benefits and product quality.

## Figures and Tables

**Table 1:** Weight percentage of ingredients to produce pasta with and without by-products.

Ingredient	CTRL [%]	Pumpkin [%]	Broccoli [%]	Artichoke [%]	Asparagus [%]
Semolina	69.00	48.09	44.12	51.52	46.86
Water	18.00	12.54	11.51	13.44	12.22
Eggs	13.00	9.06	8.31	9.71	8.83
By-product	0.00	19.89	23.66	14.19	21.05
Water to hydrate by-product	0.00	10.38	12.34	11.10	10.98
CMC	0.00	0.05	0.05	0.05	0.05

CTRL—control sample; CMC—carboxymethyl cellulose.

**Table 2:** Weight percentage of ingredients to produce gnocchi with and without by-products.

Ingredient	CTRL [%]	Asparagus [%]	Pumpkin [%]	Broccoli [%]	Artichoke [%]
Water	57.41	34.16	37.86	41.94	42.09
Potato flakes	6.32	3.76	4.68	4.61	4.98
Wheat flour	25.84	15.37	19.14	18.87	20.36
Potato starch	9.19	5.47	6.81	6.71	7.24
Rice flour	0.00	0.00	0.00	0.00	0.00
Salt	0.72	0.72	0.53	0.52	0.57
Lactic acid	0.43	0.43	0.32	0.31	0.34
Sorbic acid	0.07	0.07	0.05	0.05	0.06
Safflower	0.03	0.03	0.02	0.02	0.02
Water to hydrate by-product	0.00	22.00	17.23	9.17	11.31
CMC	0.00	0.00	0.17	0.17	0.00
By-product	0.00	18.00	13.19	17.61	13.03

CTRL—control sample; CMC—carboxymethyl cellulose.

**Table 3:** Water content expressed as weight percentage of fresh and dehydrated by-products, using both the lab-scale and the industrial dehydration system.

By-Product		Water Content [%]	
		Fresh	Dried
Pumpkin	Lab-scale	86.9 ± 0.7 ^b^	4.1 ± 0.6 ^e^
	Industrial		7.1 ± 0.6 ^c^
Artichoke	Lab-scale	86.6 ± 1.6 ^b^	5.0 ± 0.4 ^d,e^
	Industrial		4.1 ± 0.6 ^e^
Asparagus	Lab-scale	92.1 ± 0.8 ^a^	9.3 ± 0.7 ^a,b^
	Industrial		11.0 ± 0.9 ^a^
Broccoli	Lab-scale	90.8 ± 1.1 ^a^	7.6 ± 0.8 ^b,c^
	Industrial		6.5 ± 0.8 ^c,d^

Data in each column with different superscript letters are statistically different (*p* < 0.05).

**Table 4:** Total phenol content (TPC), total flavonoid content (TFC), antioxidant activity (ABTS and FRAP assays), and total dietary fiber (TDF) of by-products and main ingredients of pasta and gnocchi.

Ingredients		TPC $\left[\frac{mg\;GAE}{g\;dw}\right]$	TFC $\left[\frac{mg\;QE}{g\;dw}\right]$	ABTS $\left[\frac{mg\;TE}{g\;dw}\right]$	FRAP $\left[\frac{\mu mol\;Fe(III)}{g\;dw}\right]$	TDF $\left[\frac{g}{100\;g}\right]$
Pumpkin	Lab	5.30 ± 0.14 ^g^	5.61 ± 0.16 ^f^	7.84 ± 0.42 ^e^	2.77 ± 0.12 ^d^	27.10 ± 1.56 ^e^
	Industrial	7.12 ± 0.22 ^f^	3.34 ± 0.39 ^f,g^	9.01 ± 0.23 ^d,e^	5.78 ± 0.72 ^d^	29.40 ± 1.70 ^e^
Artichoke	Lab	31.88 ± 0.39 ^b^	44.53 ± 1.18 ^a^	23.30 ± 0.39 ^b^	135.17 ± 5.93 ^a^	44.8 ± 2.58 ^c^
	Industrial	34.88 ± 1.05 ^a^	40.02 ± 0.20 ^b^	26.94 ± 0.69 ^a^	144.09 ± 5.92 ^a^	65.4 ± 3.76 ^b^
Asparagus	Lab	22.66 ± 0.53 ^c^	16.71 ± 0.53 ^c.d^	16.90 ± 0.47 ^c^	42.31 ± 7.74 ^b^	27.80 ± 1.60 ^e^
	Industrial	20.87 ± 0.37 ^d^	17.19 ± 0.59 ^c^	16.63 ± 0.95 ^c^	47.82 ± 0.59 ^b^	33.1 ± 1.91 ^d.e^
Broccoli	Lab	14.25 ± 0.46 ^e^	11.89 ± 1.08 ^e^	9.55 ± 0.52 ^d^	16.58 ± 0.38 ^c^	38.90 ± 2.24 ^c,d^
	Industrial	14.00 ± 0.13 ^e^	14.74 ± 2.09 ^d^	8.78 ± 0.39 ^d,e^	16.88 ± 0.52 ^c^	39.1 ± 2.25 ^c,d^
Pasta	Semola	2.38 ± 0.17 ^h^	2.34 ± 0.13 ^g,h^	3.36 ± 0.26 ^f,g^	0.83 ± 0.09 ^d^	3.70 ± 0.22 ^f^
	Uovo	2.14 ± 0.07 ^h^	2.35 ± 0.37 ^g,h^	2.32 ± 0.36 ^g^	0.95 ± 0.07 ^d^	<LQ
	CMC	0.13 ± 0.01 ^i^	0.46 ± 0.15 ^h^	0.27 ± 0.03 ^h^	0.07 ± 0.05 ^d^	75.50 ± 4.34 ^a^
Gnocchi	Fiocco di patate	1.88 ± 0.17 ^h^	1.41 ± 0.04 ^g,h^	3.42 ± 0.39 ^f,g^	4.59 ± 0.28 ^d^	5.0 ± 0.3 ^f^
	Farina	2.35 ± 0.09 ^h^	1.79 ± 0.36 ^g,h^	3.92 ± 0.80 ^f^	0.25 ± 0.02 ^d^	3.50 ± 0.21 ^f^
	Fecola di patate	0.21 ± 0.01 ^i^	0.66 ± 0.08 ^h^	0.49 ± 0.02 ^h^	0.22 ± 0.19 ^d^	<LQ

Data in each column with different superscript letters are statistically different (*p* < 0.05).

**Table 5:** Total phenol content (TPC), total flavonoid content (TFC), antioxidant activity (ABTS and FRAP assays), and total dietary fiber (TDF) of pasta and gnocchi samples.

Samples	TPC $\left[\frac{mg\;GAE}{g\;dw}\right]$			TFC $\left[\frac{mg\;QE}{g\;dw}\right]$			ABTS $\left[\frac{mg\;TE}{g\;dw}\right]$			FRAP $\left[\frac{\mu mol\;Fe(III)}{g\;dw}\right]$			TDF $\left[\frac{g}{100\;g}\right]$			
	M	P	$\bar{E}\%$	M	P	$\bar{E}\%$	M	P	$\bar{E}\%$	M	P	$\bar{E}\%$	M	P	$\bar{E}\%$	
P-CTRL	1.98 ± 0.15 ^f^	2.56	29.3	1.35 ± 0.09 ^d^	2.53	87.3	4.57 ± 1.01 ^b,c,d^	3.58	21.6	0.71 ± 0.11 ^f^	0.71	27.1	3 ± 0.183 ^f^	2.55		14.9
P-Broccoli	5.12 ± 0.18 ^c,d^	6.68	30.5	4.20 ± 0.18 ^c^	6.93	65.0	5.93 ± 0.85 ^b,c^	5.47	7.7	10.98 ± 1.52 ^d^	6.64	39.5	9.7 ± 0.566 ^b,c^	10.9		12.6
P-Artichoke	5.66 ± 0.74 ^c^	9.79	72.9	7.08 ± 1.26 ^b^	10.91	54.1	8.71 ± 1.18 ^a^	8.82	1.2	25.52 ± 0.76 ^b^	32.84	28.7	14.5 ± 0.841^a^	11.2		22.6
P-Asparagus	4.95 ± 0.24 ^c,d^	8.45	70.7	4.27 ± 0.24 ^c^	7.25	69.8	6.28 ± 0.27 ^b^	7.80	24.2	12.06 ± 1.31 ^d^	15.94	32.2	9.7 ± 0.566 ^b,c^	8.74		9.9
P-Pumpkin	3.35 ± 0.15 ^e^	3.92	16.9	2.18 ± 0.23 ^d^	3.13	43.6	5.31 ± 0.45 ^b,c^	5.20	2.2	2.64 ± 0.27 ^e,f^	2.36	10.7	8.1 ± 0.479 ^d^	7.66		5.4
G-CTRL	2.04 ± 0.09 ^f^	1.52	25.4	2.22 ± 0.13 ^d^	1.25	43.7	1.97 ± 0.11 ^e^	2.60	32.1	0.62 ± 0.02 ^f^	0.76	23.1	2.4 ± 0.148 ^f^	1.22		49.2
G-Broccoli	7.49 ± 0.47 ^b^	5.73	23.6	6.75 ± 0.45 ^b^	5.79	14.2	4.33 ± 0.13 ^c,d^	4.71	8.8	15.37 ± 0.83 ^c^	6.17	59.8	9.8 ± 0.572 ^b,c^	7.78		20.6
G-Artichoke	10.5 ± 0.28 ^a^	9.97	5.01	9.05 ± 0.53 ^a^	11.07	22.3	4.96 ± 0.30 ^b,c,d^	8.77	76.9	41.52 ± 0.63 ^a^	37.06	10.7	11.0 ± 0.641 ^b^	9.49		13.8
G-Asparagus	8.09 ± 0.25 ^b^	9.04	11.8	7.35 ± 0.71 ^b^	7.45	1.3	4.94 ± 0.14 ^b,c,d^	8.08	63.5	14.65 ± 0.73 ^c^	19.01	29.8	8.7 ± 0.509 ^c,d^	6.68	23.2	
G-Pumpkin	4.38 ± 0.17 ^d^	3.08	29.6	2.46 ± 0.28 ^d^	2.18	11.3	3.28 ± 0.19 ^d,e^	4.41	34.5	4.19 ± 0.33 ^e^	2.15	48.7	5.4 ± 0.320 ^e^	4.78	11.5	

Data in each column with different superscript letters are statistically different (*p* < 0.05). $\bar{E}\%$— mean relative deviation modulus; M—measured value; P—predicted value. P-CTRL—control pasta; P-Broccoli—pasta with broccoli by-products; P-Artichoke—pasta with artichoke by-products; P-Asparagus—pasta with asparagus by-products; P-Pumpkin—pasta with pumpkin by-products. G-CTRL—control gnocchi; G-Broccoli—gnocchi with broccoli by-products; G-Artichoke—gnocchi with artichoke by-products; G-Asparagus—gnocchi with asparagus by-products; G-Pumpkin—gnocchi with pumpkin by-products.

## Data Availability

The original contributions presented in the study are included in the article; further inquiries can be directed to the corresponding author.
